# Virtual Reality in Cognitive Rehabilitation: A Pilot Study on Usability and Preliminary Clinical Outcomes in Seniors with Mild Cognitive Impairment

**DOI:** 10.1089/jmedxr.2024.0051

**Published:** 2025-03-21

**Authors:** Jose Ferrer Costa, Maria Jose Ciudad, Maribel González Abad, José Luis Rodríguez García

**Affiliations:** ^1^ Innovation and Projects Department, Badalona Serveis Assistencials, Badalona, Spain.; ^2^ Research Support Unit, Metropolitana Nord, Jordi Gol i Gurina University Institute for Primary Health Care Research Foundation (IDIAPJGol), Mataró, Spain.; ^3^ Geriatrics and Palliative Care Service, El Carme Intermediate Care Center, Badalona Serveis Assistencials, Badalona, Spain.; ^4^ Department of Clinical Psychology and Psychobiology, Faculty of Psychology, Universitat de Barcelona, Barcelona, Spain.

**Keywords:** virtual reality, mild cognitive impairment, cognitive rehabilitation, usability, acceptability, geriatric health

## Abstract

With the global population rapidly aging, cognitive decline, including mild cognitive impairment (MCI), is becoming increasingly common. MCI impacts essential cognitive functions like memory and attention, significantly reducing the quality of life for those affected and increasing the burden on healthcare systems. Virtual reality (VR) has emerged as a promising tool for enhancing cognitive rehabilitation by creating immersive and engaging environments. This pilot study aimed to evaluate the usability, acceptability, and preliminary cognitive benefits of VR-based cognitive stimulation exercises for older adults with MCI. The intervention consisted of eight VR and augmented reality sessions over 4 weeks, integrating interactive tasks and 360-degree videos tailored to enhance cognitive flexibility, visuospatial processing, and memory. Forty-five participants enrolled, with 40 completing the full intervention and final postevaluation, and usability feedback was collected from 42 participants, including two who completed partial sessions. Usability was assessed using an adapted System Usability Scale (SUS). Patients rated the VR content (81.9) and headset usability (86.5) highly reflecting strong engagement and comfort. Four healthcare professionals also provided a high SUS score (85.2), emphasizing the program’s clinical relevance and feasibility for integration into rehabilitation workflows. Cognitive assessments indicated a modest improvement in Trail Making Test A scores (*p* = 0.037), which may suggest enhanced cognitive flexibility. Other measures, including Mini-Mental State Examination, Montreal Cognitive Assessment, Digit Span, and Symbol Digit Modalities Test, showed trends toward improvement but did not reach statistical significance. These exploratory findings align with the short duration of the intervention and the study’s feasibility focus. The iterative, user-centered design process ensured the intervention was accessible, safe, and adaptable to participants’ needs and clinical workflow. These findings suggest that VR-based cognitive stimulation programs are highly usable and acceptable and show potential for improving cognitive performance in older adults with MCI. Further research with larger sample sizes and longer intervention periods is warranted to evaluate clinical impact and functional outcomes.

## Introduction

As the global population continues to age rapidly, the associated rise in age-related cognitive decline presents an urgent challenge for health care systems worldwide. The number of people aged 65 years and older is expected to rise from an estimated 727 million in 2020 to more than double by 2050, reaching over 1.5 billion, according to the World Health Organization.^1^

In Spain, this global trend is mirrored by a rapidly aging population. Recent data from the Instituto Nacional de Estadística and the Ministry of Health indicate an accelerated aging trend, posing substantial challenges for healthcare services. The aging index reached 142.3% in 2024, indicating 142 individuals aged 65 years or older for every 100 under the age of 16. By 2050, projections estimate that 30.4% of the population will be over 65 years, underscoring the critical need for effective age-related interventions. The centenarian population is also projected to keep rising, from 14,287 currently to 226,932 by 2072, further emphasizing the need for effective interventions to address age-related conditions.^2^

This demographic shift is accompanied by a growing prevalence of cognitive decline, with Alzheimer’s disease (AD) alone expected to affect 139 million people globally by 2050.^3^ AD currently affects ∼5% of individuals over 65 years in Spain, with around 800,000 diagnosed cases. This number is anticipated to double in the coming decades, potentially affecting over 41 out of every 1,000 people by 2050.^4^ Cognitive impairments, including memory loss, diminished attention, and executive dysfunction, significantly impact the quality of life for affected individuals and impose a heavy burden on caregivers and health care systems.^3–5^

In response to this growing challenge, the Spanish Ministry of Health emphasizes the importance of cognitive rehabilitation programs and the use of technology in managing these conditions. The plan advocates for activity and cognitive stimulation programs tailored to the neuropsychological characteristics of different disease stages, prioritizing these interventions from the early phases of the disease. Additionally, it highlights the need to define and standardize the professional profile required for the design and implementation of these programs and to enhance awareness and training among health care professionals, patients, and caregivers regarding the available cognitive rehabilitation options.^5,6^

In this context, virtual reality (VR) has emerged as a promising tool for cognitive stimulation in individuals with cognitive impairment.^7^ VR technology stands out by providing immersive, engaging environments that replicate real-life scenarios safely, making it a promising tool for cognitive rehabilitation. Numerous studies have demonstrated that VR can significantly enhance cognitive skills and reduce distractions in individuals with mild cognitive impairment (MCI).^7–14^ A recent meta-analysis by Perra et al.^14^ further confirms the positive impact of VR-based cognitive rehabilitation programs, showing improvements not only in cognitive functions and daily functioning but also in depressive symptoms and quality of life.

Building on these foundations, our pilot study investigates the usability and acceptability of a VR-based cognitive stimulation program for older adults with MCI. This article outlines the development and evaluation of the program, which was created through collaboration between health care professionals and technical experts. The study seeks to provide initial insights into the potential integration of VR interventions in clinical settings.

## Materials and Methods

### Study design

This nonrandomized, prospective pilot study evaluated a four-week VR-based cognitive stimulation program at Centro Sociosanitario El Carme, a daycare clinic offering cognitive rehabilitation. The intervention integrated seamlessly into patients’ existing schedules of 45-min sessions, conducted two to three times weekly. Ethical approval was obtained (CEIC HGTiP, protocol PI-23-195), and the study is registered on ClinicalTrials.gov (NCT06155721).

### Recruitment and inclusion criteria

Recruitment was conducted from the total pool of patients enrolled in the cognitive rehabilitation program at our service. Recruitment began in October 2023, following an informational session with potential participants. From a total pool of 64 patients enrolled in the rehabilitation program, 53 met the inclusion criteria based on their clinical evaluations conducted at admission. These criteria included age over 60 years and a Mini-Mental State Examination (MMSE) score >23. Among the eligible patients, 45 expressed interest, provided informed consent, and were included in the study.

Prior to the intervention, participants completed a pre-evaluation battery of cognitive assessments, which included a repeat MMSE. Exclusion criteria for the study included severe psychiatric disorders, epilepsy, significant sensory impairments, or a history of motion sickness.

To accommodate the high number of eligible participants, the intervention was conducted in two cohorts. In total, 45 participants were included, 42 of whom provided usability feedback, and 40 completed the clinical postevaluation. Dropouts included two participants who discontinued due to cybersickness and three who did not complete the postevaluation protocols (details on exclusion in the Results section).

### Intervention

**FIG. 1.**
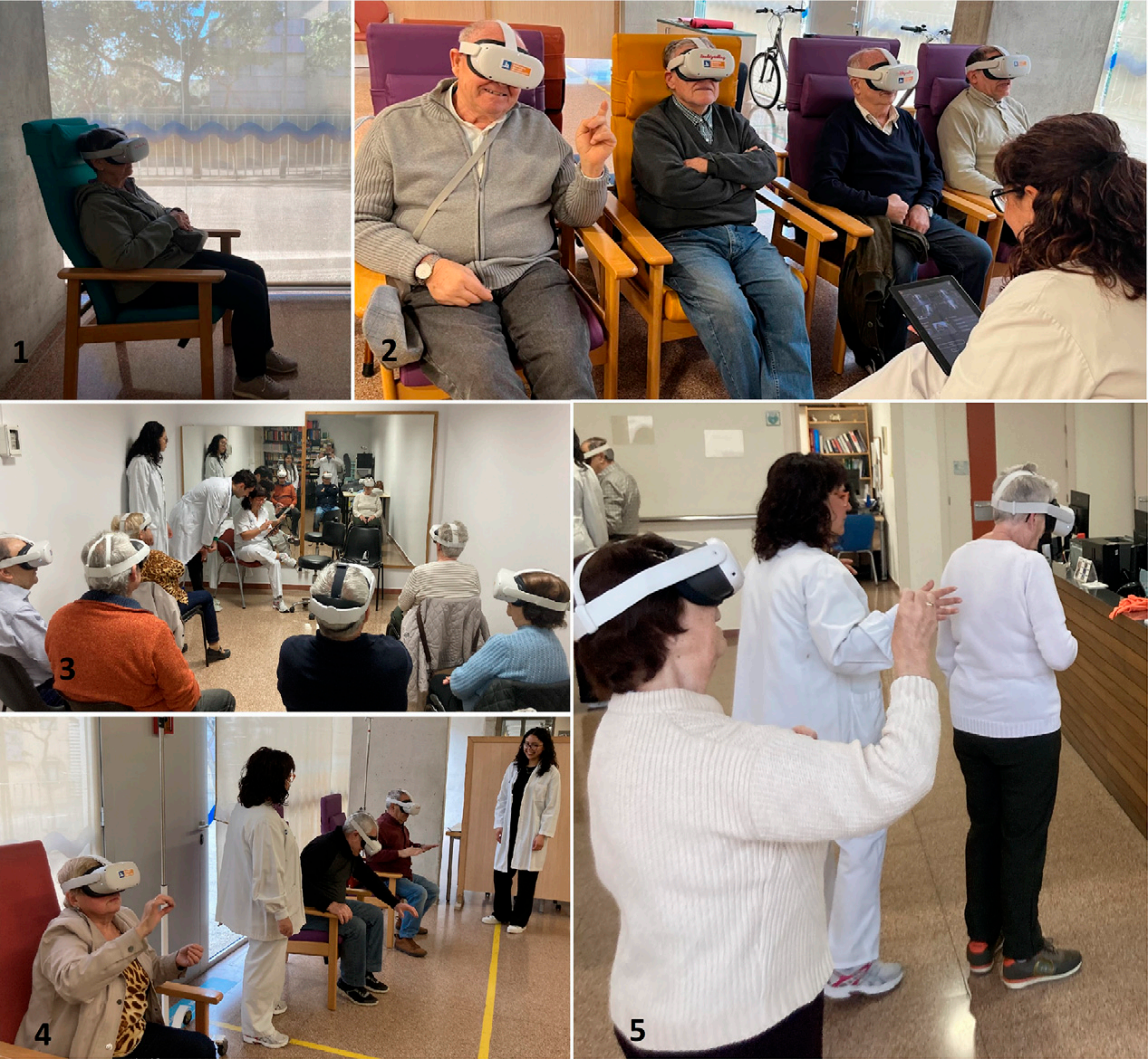
Patient engagement in VR-based cognitive stimulation sessions. Images showcase older adults with MCI actively participating in the VR intervention at Centro Sociosanitario El Carme. **(1)** Initial familiarization phase with mindfulness meditation, introducing participants to the VR environment. **(2, 3)** Guided 360-degree video sessions, designed to enhance memory, and attention while fostering social interaction between participants. **(4, 5)** Interactive VR environments demonstrating task adaptability for seated or standing positions, ensuring accessibility and accommodating diverse physical abilities. MCI, mild cognitive impairment; VR, virtual reality.

The 4-week program included eight VR sessions, lasting 7–20 min, integrated into the existing cognitive rehabilitation framework. Content complexity increased progressively across three stages to optimize cognitive stimulation and participant adaptation (Fig. 1).

The VR intervention was conducted using Oculus Quest 2 and Quest 3 headsets. The Oculus Quest 2, featuring a black-and-white passthrough, was primarily used by participants for various VR content, including the virtual supermarket, 360-degree videos, and the meditation session. One Oculus Quest 3 headset, offering color passthrough, was also available and used by some participants during certain sessions. All exercises were designed to function seamlessly across both devices, ensuring consistency in the intervention.

The intervention was developed following core principles, including gradual adaptation, high-tech and high-touch integration, and iterative user-centered design, as summarized in Table 1.

**FIG. 2.**
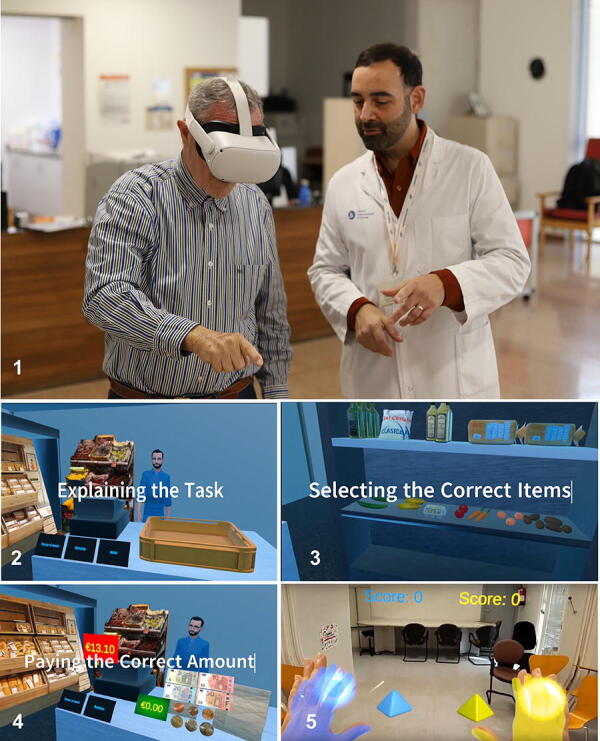
Interactive virtual and augmented reality exercises for cognitive and functional stimulation. Images demonstrate participants and tasks from the VR intervention at Centro Sociosanitario El Carme. Therapist guiding a participant **(1)** through a fully immersive virtual environment **(2)**. VR tasks simulating real-world scenarios, including selecting the correct items in a virtual supermarket **(3)** and paying the correct amount at checkout **(4)**. AR-based spatial orientation and movement exercises **(5)**, designed to enhance accessibility with options for seated or standing interaction. AR, augmented reality; VR, virtual reality.

1. **Initial Familiarization: Guided Mindfulness Meditation**
  The first session introduced participants to the VR headset and hand-tracking technology in a calm setting using a guided mindfulness meditation. This approach alleviated apprehension, building confidence for more interactive experiences.
2. **Group VR Sessions: 360-Degree Videos**
  Participants engaged in 360-degree immersive videos featuring local landmarks and notable sites, such as the Barcelona Aquarium, delivered via the Reality Telling platform. Small groups (3–6 participants) encouraged shared experiences and social interaction. Therapists controlled the headsets via a synchronized tablet and guided attention with prompts like “What building are we passing?” while participants shared related memories, stimulating episodic memory, and reminiscence.
  Sessions began with stationary videos to minimize motion sickness, progressing to guided movements through familiar locations, enhancing visuospatial perception and sustained attention. After the video sessions, the therapist encouraged participants to discuss observed details, reinforcing memory, and consolidating their experiences.
3. **Individual Interactive Sessions: Three-Dimensional Virtual Environments**
  Participants engaged in immersive three-dimensional VR environments for tasks such as shopping and calculating totals at a virtual checkout. Augmented reality (AR)-based exercises included puzzles where participants manipulated virtual geometric shapes to match colors and positions, resembling Tetris-like tasks. Additionally, dynamic object interactions required participants to touch virtual items that shifted position or transformed upon collision. These activities targeted visuospatial skills, cognitive flexibility, and motor coordination, with tasks adaptable for seated or standing positions to ensure accessibility and safety (Fig. 2).

**Table 1.** Intervention Design Principles

Principle	Purpose	Key actions	Impact
Gradual adaptation and progressive exposure	To ease participants into the VR environment and sustain engagement.	Started with passive experiences (e.g., mindfulness) and progressed to interactive, immersive tasks.	Minimized cybersickness, built confidence, and encouraged active participation.
High-tech, high-touch integration	To balance advanced technology with personalized therapeutic care.	Therapists provided real-time feedback, encouragement, and guided interaction with VR content.	Enhanced patient-centered care, fostering trust, and maximizing therapeutic outcomes.
Safety integration	To ensure participant well-being and reduce potential risks during sessions.	Therapists trained to detect discomfort; tasks adjusted to prevent falls (Fig. 4)	Maintained a secure environment, enabling older adults to fully engage without physical strain.
User-centered and iterative design	To refine the program based on participant and therapist feedback.	Collected open-ended feedback to improve clarity, usability, and task design (e.g., clearer instructions).	Ensured the program remained adaptable, engaging, and accessible for diverse needs.

This table outlines the four core principles that guided the development and implementation of the VR-based cognitive stimulation intervention. Each principle is described with its specific purpose, the key actions undertaken to address it, and the resulting impact on participants and the overall effectiveness of the program. The iterative approach ensured the intervention was user-centered, safe, and therapeutically effective.

VR, virtual reality.

### Safety and support

Therapists closely monitored participants to address discomfort and ensure safety, providing real-time support. Postsession debriefings facilitated reflection on the session’s content and gathered valuable feedback to refine the intervention.

### Outcome measures

#### Primary outcomes: Usability and acceptability

The usability and acceptability of the VR intervention were assessed using an adapted version of the original System Usability Scale (SUS), developed by Brooke.^15,16^ This widely used tool evaluates the usability of a range of systems on a scale from 0 to 100, where scores above 68 are considered above average. The adaptation to an 8-item format was made to better suit the older adult population with MCI, reducing respondent fatigue while retaining the scale’s robustness and core usability evaluation framework.

The scoring methodology for the adapted SUS remained consistent with the original framework, ensuring comparability despite the reduced number of items. Each response was scored based on its phrasing. For positively worded items, the raw score was adjusted by subtracting 1, while for negatively worded items, the adjustment involved subtracting the raw score from 5. These adjustments ensured that higher scores consistently indicated better usability.

Once all eight items were adjusted, their scores were summed to calculate a total raw score. To normalize this raw score to a 0–100 scale, the total was multiplied by 100/(4 × *N*), where *N* = 8 represents the number of items in the adapted scale. This scaling approach preserved the interpretability of the results within the standard SUS framework, allowing for direct comparison with studies utilizing the original 10-item scale.

Although the 8-item adaptation retained the core framework of the original SUS and ensured consistent scoring, it has not been independently validated. This limitation is noted and discussed further in the Limitations section.

The SUS was administered in two formats:

- SUS for patients: Two separate SUS assessments were conducted. The first focused on the usability of the VR content, evaluating clarity, complexity, and overall user experience. The second assessed the physical comfort and usability of the Oculus Quest 2 and 3 headsets, including factors such as weight, fit, and potential discomforts like cybersickness. The eight questions from both SUS evaluations are detailed in the results section.
- SUS for professionals: Evaluated clinical integration and usability, capturing healthcare professionals’ perspectives on patient usability and workflow relevance.

### Secondary outcomes: Cognitive function

Cognitive performance was assessed using a standardized neuropsychological battery administered pre- and postintervention. This battery included:

- MMSE: A general cognitive screening tool.
- Montreal Cognitive Assessment (MoCA): Evaluating multiple cognitive domains, including memory, attention, and executive function.
- Digit Span Tests (Direct and Inverse): Measuring working memory and attention.
- Trail Making Test A (TMTA): A task-switching test assessing visual attention and cognitive flexibility.
- Symbol Digit Modalities Test (SDMT): Evaluating processing speed and visuospatial functions.

### Statistical analysis

The data analysis was conducted using a structured approach, focusing first on the usability and acceptability measures and then on the clinical variables:

- **SUS:** Scores for patients and professionals were normalized to a 0–100 scale following Brooke’s methodology.^15,16^ Scores above 68 were interpreted as above average, and scores closer to 100 reflect higher levels of usability and user satisfaction.
- **Pre–post comparisons:** Paired *t*-tests evaluated changes in cognitive test scores (MMSE, MoCA, TMTA, Digit Span, and SDMT) pre- and postintervention.
- **Effect size:** Cohen’s *d* provided an understanding of the magnitude of change in cognitive outcomes.
- **Correlation analysis:** Pearson’s coefficients assessed the strength of the relationship between pre- and postintervention scores. Statistical significance was set at *p* < 0.05.

## Results

### Sample demographics

The study initially included 45 participants, of whom 40 completed the full intervention and final postevaluation. The demographic characteristics of all 45 participants are presented in Table 2, along with a note on those who did not complete the study. The average age of the participants was 75 years (±5.9), with 27 (60%) identifying as females and 18 (40%) as males.

**Table 2.** Demographic Characteristics of the Study Participants

Demographic characteristics	Total included *n* (%)	Completed intervention *n* (%)	Excluded
Total participants	45 (100%)	40 (89%)	
Age (years)	75 ± 5.9 (mean/SD)	75.2 ± 6.2 (mean/SD)	
Sex			
Female	27 (60%)	23 (57.5%)	4
Male	18 (40%)	17 (42.5%)	1

SD, standard deviation.

Five participants did not fully complete the study. One participant completed all eight intervention sessions but was unable to attend the final postevaluation due to relocating to another area. Another participant completed six sessions but discontinued due to cybersickness. These two participants were included in the usability analysis but excluded from the clinical outcomes. Three participants were excluded: one after five sessions due to eye surgery, one after four sessions due to cybersickness, and one after two sessions due to discharge from the rehabilitation program. Neither provided postevaluation or SUS data (see Table 3 for details).

**Table 3.** Exclusion Details and Participation in Evaluations

Reason for exclusion	Age (years)	Sex	Sessions completed	Participated in SUS	Participated in clinical outcomes
Cybersickness	75	Female	6	Yes	No
Discharged (relocated to another area)	78	Male	8	Yes	No
Discharged (relocated to another area)	80	Female	2	No	No
Eye surgery	75	Female	4	No	No
Cybersickness	72	Female	4	No	No

SUS, System Usability Scale.

### System Usability Scale for patients

The usability and acceptability of the VR intervention were assessed using two separate SUS evaluations for patients: one focusing on the usability of the VR content and another on the physical comfort and usability of the VR headsets. A total of 42 participants completed the SUS evaluations, including two participants who did not complete the postevaluation but participated in the usability analysis after completing six and eight sessions, respectively. The results are summarized in Table 4.

**Table 4.** Patient Responses to the System Usability Scale

System Usability Scale (SUS) for patients	Strongly disagree (%)	Disagree (%)	Neutral (%)	Agree (%)	Strongly agree (%)
Evaluation of the simulation–Usability scale of the system–Total Score of 81.925					
”I think I would like to use this program frequently.”	0.00	4.76	4.76	14.29	76.19
”I think the program is too complex.”	57.14	11.90	9.52	19.05	2.38
”I think that exercises with glasses are more entertaining than in a conventional way.”	14.29	7.14	28.57	16.67	33.33
”I think the simulation options are clear and well integrated.”	0.00	0.00	0.00	19.05	80.95
”I think some options are difficult to follow.”	59.52	7.14	7.14	14.29	11.90
”I think people will be able to learn how to use this system easily.”	0.00	2.38	16.67	26.19	54.76
”I felt comfortable using this system.”	2.38	4.76	0.00	2.38	90.48
”I had to learn many things before I could use the system.”	73.81	2.38	9.52	11.90	2.38
Valuation of the VR glasses—Meta Quest 2 and 3—Total Score of 86.5					
”The glasses were very heavy and uncomfortable”	64.29	9.52	2.38	14.29	9.52
”The use of glasses and controls has been very complicated”	64.29	9.52	19.05	4.76	2.38
”I have felt fatigue in my arms and fingers”	92.86	2.38	2.38	2.38	0.00
”I felt visual discomfort”	83.33	2.38	2.38	9.52	2.38
”I felt dizzy”	73.81	2.38	2.38	19.05	2.38
”I have felt a headache”	95.24	0.00	2.38	2.38	0.00
”I had to stop the simulation because of the discomforts referred to”	90.48	2.38	4.76	2.38	0.00
”I think it would be comfortable to use these glasses for a long time”	4.76	9.52	11.90	28.57	45.24

This table summarizes the responses to two System Usability Scale evaluations for the VR intervention: one focusing on the usability of the VR content (total score: 81.925) and another assessing the physical comfort and usability of the VR headsets (total score: 86.5). It captures the experiences of the patient participants (*n* = 42), including ease of use, comfort, entertainment value, and potential challenges, such as dizziness or control difficulties. (Translated from the Spanish version used in the study.)

The SUS for VR content obtained a high usability score of 81.9, indicating that participants found the immersive experience engaging and easy to navigate. A substantial majority (90.48%) reported feeling comfortable using the system, and 76.19% expressed a strong desire to use the program frequently. Only a small proportion (21.43% agree and strongly agree) found the program to be complex.

The VR headsets were rated with an even higher SUS score of 86.5, reflecting overall comfort and usability. Most participants (64.29%) disagreed that the headsets were heavy or uncomfortable, and 95.24% did not experience headaches during use. However, 21.43% (agree and strongly agree) reported experiencing some degree of dizziness or cybersickness, and a small number found the controls challenging to use.

Qualitative feedback reinforced these findings, with participants frequently mentioning the entertainment value and engagement of the VR exercises. Notably, underwater scenes were highlighted as particularly enjoyable. Some participants acknowledged that the VR activities were complementary to conventional cognitive exercises, which they found more cognitively demanding. Minor issues, such as discomfort when wearing corrective glasses with the VR headset, were also noted.

### System Usability Scale for professionals

The usability of the VR simulation content as assessed by healthcare professionals is summarized in Table 5. The SUS evaluation included feedback from a neuropsychologist, a nurse, and two nurse assistants who participated in the implementation of the sessions with patients. The overall SUS score for the program was 85.2, indicating high usability. Specifically, all of the professionals strongly agreed that the program could improve the quality of patient care and reported feeling comfortable using the system for patient care.

**Table 5.** Professional Responses to the System Usability Scale

System Usability Scale (SUS) for professionals					
Evaluation of the simulation—total score of 85.2	Strongly disagree (%)	Disagree (%)	Neutral (%)	Agree (%)	Strongly agree (%)
“I believe this program will help improve the quality of patient care.”	0.00	0.00	0.00	0.00	100.00
“I think the program interface is intuitive for the user.”	0.00	0.00	0.00	50.00	75.00
“I believe that the program can be easily integrated into daily care practice.”	0.00	0.00	0.00	0.00	100.00
“I believe that the functions and features of simulation are clear and relevant to patient care.”	0.00	0.00	0.00	25.00	75.00
“Some of the functionality of this system may be difficult for patients to understand or use.”	0.00	0.00	25.00	50.00	25.00
“I believe that patients will be able to learn to use this system easily.”	0.00	25.00	0.00	0.00	75.00
“I have felt comfortable using this system for patient care.”	0.00	0.00	0.00	0.00	100.00
“I have needed additional time to learn how to use this system correctly.”	0.00	50.00	0.00	25.00	0.00

This table presents the responses to the System Usability Scale questions for the VR simulation content as evaluated by health care professionals (*n* = 4). It summarizes their perceptions of usability, ease of integration into patient care, and the system’s overall impact on quality of care.

Regarding the system’s intuitiveness, 75% of professionals agreed that the interface was intuitive for the user. Furthermore, 75% of professionals expressed concerns that certain functionalities might be difficult for patients to understand or use. Despite these points, 100% of professionals agreed that the features of the simulation were clear and relevant to patient care, and 75% felt confident that patients would be able to learn to use the system easily.

### Clinical variables

The analysis of cognitive performance pre- and postintervention was conducted using paired *t*-tests and correlation coefficients for the standardized cognitive assessments. The detailed statistical outcomes are presented below.

- **MMSE:** The mean scores showed no significant change pre- to postintervention [*t*(39) = −1.14, *p* = 0.131, Cohen’s *d* = −0.18]. The correlation between pre- and postscores was moderate (*r* = 0.43, *p* = 0.006).
- **MoCA:** The mean scores did not differ significantly from pre- to postintervention [*t*(39) = −0.73, *p* = 0.235, Cohen’s *d* = −0.12]. A strong correlation was observed between pre- and postscores (*r* = 0.75, *p* < 0.001).
- **Digit Span Test (Direct):** No significant improvement was found [*t*(39) = 1.24, *p* = 0.889, Cohen’s *d* = 0.2], though the correlation was moderate (*r* = 0.53, *p* < 0.001).
- **Digit Span Test (Inverse):** There was no significant change [*t*(39) = −0.78, *p* = 0.22, Cohen’s *d* = −0.12], but a moderate correlation existed between pre- and postscores (*r* = 0.59, *p* < 0.001).
- **TMTA:** A statistically significant improvement was observed [*t*(39) = 1.84, *p* = 0.037, Cohen’s *d* = 0.29], with a strong correlation between pre- and postscores (*r* = 0.82, *p* < 0.001).
- **SDMT:** No significant change was detected [*t*(39) = −0.98, *p* = 0.166, Cohen’s *d* = 0.16], though a strong correlation was noted (*r* = 0.79, *p* < 0.001).

**FIG. 3.**
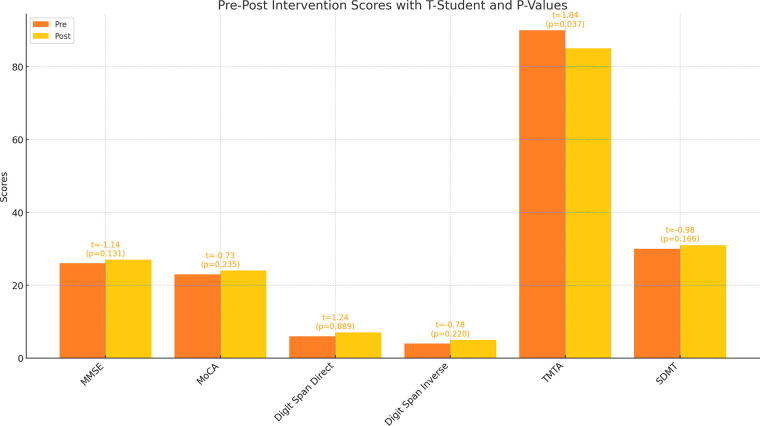
Pre- and postmedian comparison of cognitive outcomes with *p*-values. This bar chart compares the median values of cognitive and functional outcomes before and after the VR intervention. The clinical variables include MMSE, MoCA, Digit Span Direct, Digit Span Inverse, TMTA, and SDMT. Preintervention medians (yellow) and postintervention medians (orange) are displayed, with *p*-values from paired *t*-tests annotated above each comparison. MMSE, MoCA, SDMT, Symbol Digit Modalities Test; TMTA, Trail Making Test.

Table 6 and Figure 3 summarize these results, providing a comprehensive overview of the cognitive performance metrics before and after the VR intervention.

**Table 6.** Pre- and Postintervention Cognitive Performance Metrics

Clinical variable	n	Correlation (*r*)	*p* (correlation)	*t*-Value	df	*p* (*t*-test)	Cohen’s *d*
MMSE Pre–MMSE Post	40	0.43	0.006	−1.14	39	0.131	−0.18
MoCA Pre–MoCA Post	40	0.75	<0.001	−0.73	39	0.235	−0.12
Digit Span Direct Pre–Post	40	0.53	<0.001	1.24	39	0.889	0.2
Digit Span Inverse Pre–Post	40	0.59	<0.001	−0.78	39	0.22	−0.12
TMTA Pre–TMTA Post	40	0.82	<0.001	1.84	39	0.037	0.29
SDMT Pre–SDMT Post	40	0.79	<0.001	−0.98	39	0.166	0.16

This table presents the cognitive performance results for 40 participants who completed the VR intervention. Variables include the Mini-Mental State Examination (MMSE), Montreal Cognitive Assessment (MoCA), Digit Span Direct and Inverse, Trail Making Test A (TMTA), and Symbol Digit Modalities Test (SDMT). For each variable, the table provides:

*n*: Number of participants included in the analysis.

Correlation (*r*): Pearson correlation coefficient between pre- and postintervention scores.

*p* (Correlation): Significance level for the correlation.

*t*-Value: Results of paired *t*-tests comparing pre- and postintervention scores.

df: Degrees of freedom for the paired *t*-tests.

*p* (*t*-test): Significance level of the paired *t*-test.

Cohen’s *d*: Effect size indicating the magnitude of the change.

TMTA scores showed significant improvement (*p* = 0.037), indicating enhanced cognitive flexibility and processing speed. Other variables demonstrated positive trends and strong correlations, suggesting consistency in cognitive performance.

## Discussion

Considering that the primary focus of this pilot study was to evaluate the usability and acceptability of VR-based cognitive stimulation in older adults with MCI, the results suggest strong feasibility for integration into cognitive rehabilitation programs. High SUS scores from patients (81.9) and professionals (85.2), along with a strong usability score for the VR headsets (86.5), highlight the intervention’s acceptability and practicality in clinical settings.

### Clinical variables

While usability and acceptability were the primary objectives, preliminary clinical outcomes were also explored. An improvement in cognitive flexibility and processing speed (TMTA; *p* = 0.037) was observed, though this result would not remain significant under multiple-comparisons correction, and the absence of a control group limits the ability to draw causal inferences. Other cognitive measures (e.g., MMSE, MoCA, SDMT) showed positive but nonsignificant trends, likely due to the short duration of the intervention.

These results align with existing research,^17–22^ indicating that VR-based interventions may complement traditional methods by engaging users and stimulating specific cognitive functions such as attention and executive function. While promising, these findings should be interpreted cautiously, given the exploratory nature of this study.

While MCI can progress to dementia, this trajectory is not uniform for all individuals. A meta-analytic review found that cognitive rehabilitation programs can yield significant improvements in neuropsychological outcomes, suggesting that targeted interventions can have a positive impact on cognitive functions in patients with MCI.^19^ Some may experience stabilization or even cognitive improvements, especially with early and appropriate interventions. Cognitive rehabilitation, including VR-based programs, has been shown to slow cognitive decline and, in certain cases, improve cognitive performance.^20^

### User-centered design, iterative improvement, and safety

**FIG. 4.**
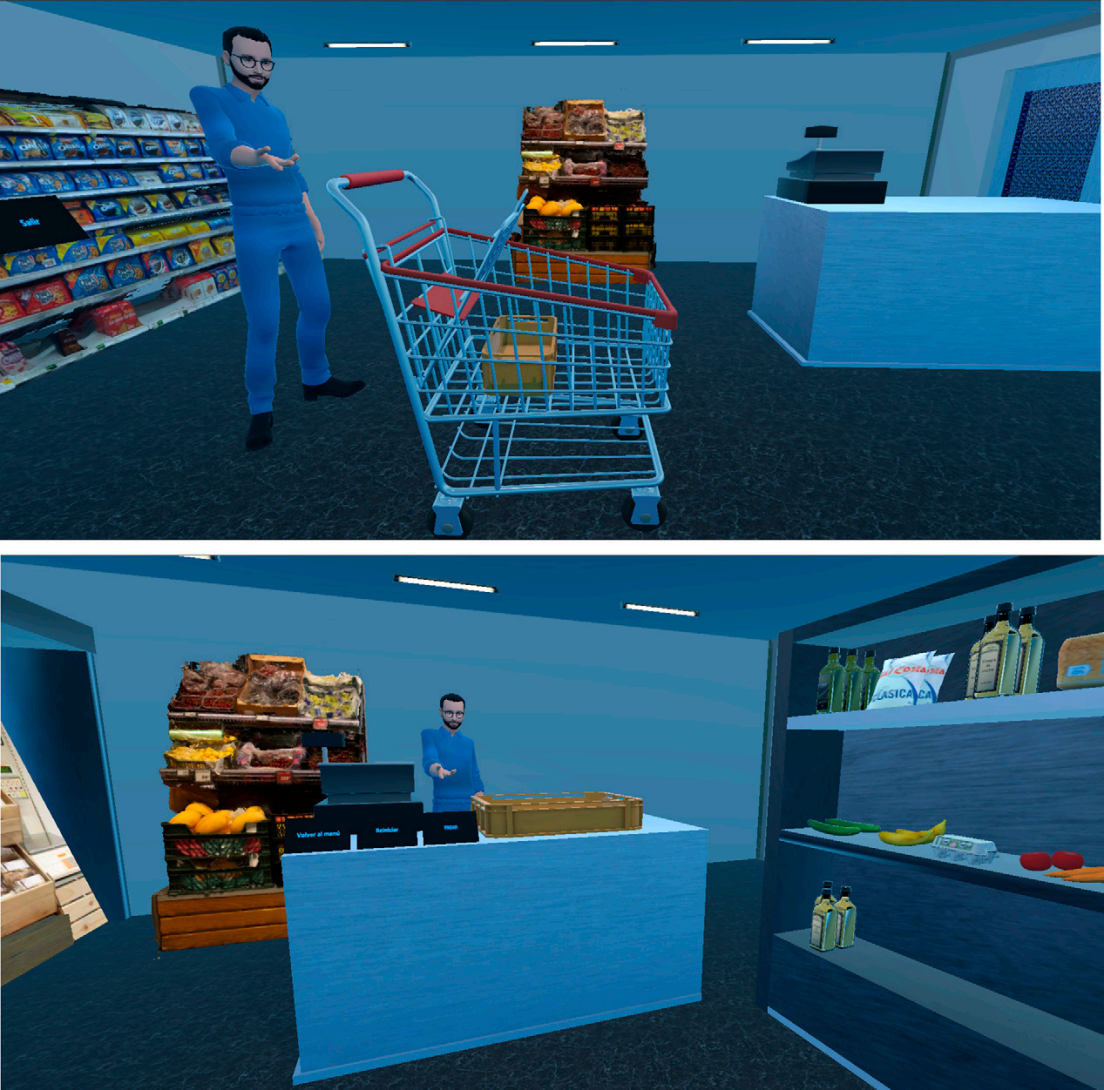
Enhancing safety and accessibility in VR shopping exercises. Images highlight modifications in the VR supermarket task to ensure participant safety. The initial design (top) required participants to bend and interact with lower shelving, presenting a fall risk. Adjustments (bottom) repositioned items and the cart to an accessible height, enabling seated or standing interaction without compromising user safety or immersion.

The intervention was developed using a user-centered, iterative design approach, ensuring accessibility and safety. Feedback from participants led to meaningful adjustments, such as modifying the virtual supermarket task to reduce fall risks for frail seniors. Initially, participants were required to bend down to place items in a virtual shopping cart, a task that posed safety challenges. Observing one participant instinctively attempt to hold onto the cart highlighted the potential risks. In response, the task was redesigned to place a virtual basket at waist height on a table, eliminating the need for bending while maintaining functionality. Figure 4 illustrates these changes, showcasing how real-world feedback can enhance both the safety and effectiveness of VR applications in therapeutic settings.

The role of therapists in monitoring participants and providing real-time support was instrumental in ensuring safety and fostering confidence during VR sessions. Personalized care, combined with advanced technology, played a pivotal role in the program’s success. Practical refinements, such as accommodations for corrective glasses and clearer instructions, further addressed usability concerns and enhanced the overall experience.

One of the notable strengths of this VR intervention was its ability to deeply engage participants. Therapists observed that patients were more willing to attempt challenging tasks within the immersive environment compared to conventional methods, such as computer-based or paper-based exercises. VR’s capacity to minimize distractions and encourage active participation highlights its potential as an engaging tool for cognitive rehabilitation.

Two participants discontinued the intervention after completing 4 and 6 sessions, respectively, due to mild symptoms of cybersickness. Although both participants were keen to continue, the decision to stop was made in agreement with the researchers, prioritizing their well-being. Despite discontinuing, they reported a positive overall experience and expressed enthusiasm for the VR intervention, suggesting that mild symptoms of cybersickness may not significantly hinder engagement for most users. This highlights the importance of careful participant monitoring to ensure safety while maintaining the intervention’s acceptability and potential for broader application.

### Limitations

This study has certain limitations that should be considered when interpreting the findings. First, the SUS was adapted to an 8-item format and translated into Spanish to improve accessibility for older adults with MCI. While this adaptation reduced respondent burden and retained the core structure of the scale, its psychometric properties remain unvalidated. To ensure comparability with the original 10-item SUS, the adjusted scoring methodology was carefully applied to normalize results to the standard 0–100 scale. Future research should focus on validating this adaptation across diverse populations to confirm its reliability and strengthen its application in VR studies.

Second, while the sample size of 45 participants is notable for a VR usability pilot, the findings may have limited generalizability. Larger, multicenter studies involving more diverse populations are recommended to further evaluate the intervention’s usability and acceptability.

Third, the intervention’s short duration—eight sessions >4 weeks—was appropriate for assessing usability but insufficient for evaluating sustained cognitive changes. Trends observed in measures such as the TMTA align with the pilot’s exploratory nature, but longer intervention periods are necessary to fully understand the potential of VR-based cognitive stimulation for cognitive and functional improvements.

Finally, as a single-arm study, the lack of a control group limits causal inferences about the intervention’s impact. While the study successfully met its primary objectives of evaluating feasibility and usability, randomized controlled trials are needed to establish clinical efficacy.

### Future research

Building upon the insights gained from this pilot, we are currently conducting a randomized controlled trial with a larger sample size, a control group, and an extended intervention period to rigorously evaluate the clinical impact of VR-based cognitive rehabilitation. This ongoing trial includes validated functional assessment tools, such as the Lawton and Brody Instrumental Activities of Daily Living Scale^23^, to assess how cognitive improvements translate into real-world functionality.

Future studies should also consider exploring subgroup analyses to determine how individuals with varying levels of cognitive impairment respond to VR interventions, enabling more personalized and targeted applications.

Additionally, research on the scalability and cost-effectiveness of VR interventions in routine clinical practice remains a key priority. Addressing challenges such as staff training, accessibility, and hardware costs will be essential to ensure that VR-based cognitive rehabilitation can be sustainably integrated into healthcare systems on a broader scale.

### Ethical considerations

The study adhered to the principles outlined in the Declaration of Helsinki and was approved by the relevant ethics committee (CEIC GTiP, protocol code PI-23-195). All participants provided written informed consent before taking part in the study. To ensure confidentiality, participants’ data were codified prior to statistical analysis, and only authorized research personnel had access to the data. Additionally, measures were taken to minimize any risk or discomfort to participants, and they were informed of their right to withdraw from the study at any point without consequence.

## Conclusions

This study highlights the potential of VR-based cognitive stimulation as a viable and engaging tool for older adults with MCI. The intervention demonstrated excellent usability, with high scores on the adapted SUS from both participants and health care professionals. Participants appreciated the immersive experience and expressed interest in continued use of the technology, while health care professionals acknowledged its practicality and relevance for integration into existing cognitive rehabilitation frameworks.

While not all observed cognitive improvements were statistically significant, the positive trends and the significant improvement in the TMTA suggest that the intervention may enhance specific cognitive domains such as attention and processing speed. These findings align with previous research indicating that VR interventions can complement traditional methods by providing an engaging and immersive format that enhances adherence and motivation.^21,22^

The real-world implementation of the study demonstrated the intervention’s adaptability to clinical settings, effectively addressing logistical challenges such as varying patient availability and staff resources. This adaptability, coupled with positive feedback, supports the feasibility of incorporating VR into cognitive rehabilitation programs.

To build on these results, future studies should incorporate larger sample sizes, longer intervention durations, and control groups to rigorously evaluate the cognitive and functional impacts of VR-based programs. Including assessments of functional abilities, such as daily living activities, would provide further insights into the practical benefits for this population. By exploring these avenues, VR has the potential to become a cornerstone in managing cognitive decline, offering a scalable and patient-centered solution for an aging population.

## Data Availability

The datasets generated or analyzed during this study can be made available from the corresponding author upon reasonable request.

## Abbreviations used

AD: Alzheimer’s disease
AR: Augmented Reality
MCI: Mild Cognitive Impairment
MMSE: Mini-Mental State Examination
MoCA: Montreal Cognitive Assessment
SDMT: Symbol Digit Modalities Test
SUS: System Usability Scale
TMTA: Trail Making Test A
VR: Virtual Reality

## References

[1] United Nations. World Population Ageing 2020 Highlights. UN: New York; 2020. Available from: https://www.un.org/development/desa/pd/news/world-population-ageing-2020-highlights [Last accessed: August 26, 2024].

[2] Instituto Nacional de Estadística. Proyecciones de Población 2022-2072 [Internet]. INE: Madrid; 2022. Available from: https://www.ine.es/ [Last accessed: March 07, 2025].

[3] World Health Organization. Dementia [Internet]. Geneva: WHO; 2023. Available from: https://www.who.int/news-room/fact-sheets/detail/dementia [Last accessed: August 26, 2024].

[4] Alzheimer’s Disease International. World Alzheimer Report 2019: Attitudes to dementia. ADI: London; 2019. Available from: https://www.alzint.org/u/WorldAlzheimerReport2019.pdf [Last accessed: August 26, 2024].

[5] Ministerio de Sanidad, Consumo y Bienestar Social. Plan Integral de Alzheimer y otras Demencias 2019-2023. Ministerio de Sanidad: Madrid; 2019. Available from: https://www.sanidad.gob.es/profesionales/saludPublica/docs/Plan_Integral_Alhzeimer_Octubre_2019.pdf [Last accessed: August 26, 2024].

[6] Instituto de Mayores y Servicios Sociales (IMSERSO). Estrategia Nacional de Personas Mayores para un Envejecimiento Activo y para su Buen Trato 2018–2021. Madrid: IMSERSO; 2018. Available from: https://www.algec.org/wp-content/uploads/2017/12/Estrateg-Nacde-PM-2018-Imserso.pdf [Last accessed: August 26, 2024].

[7] Bauer AC, Andringa G. The potential of immersive virtual reality for cognitive training in elderly. Gerontology 2020;66(6):614–623; doi: 10.1159/000509830 32906122

[8] Liao YY, Chen IH, Lin YJ, et al. Effects of virtual reality-based physical and cognitive training on executive function and dual-task gait performance in older adults with mild cognitive impairment: A randomized control trial. Front Aging Neurosci 2019;11:162; doi: 10.3389/fnagi.2019.00162 31379553 PMC6646677

[9] Gómez-Cáceres B, Cano-López I, Aliño M, et al. Effectiveness of virtual reality-based neuropsychological interventions in improving cognitive functioning in patients with mild cognitive impairment: A systematic review and meta-analysis. Clin Neuropsychol 2023;37(7):1337–1370; doi: 10.1080/13854046.2022.2148283 36416175

[10] Muñoz J, Mehrabi S, Li Y, et al. Immersive virtual reality exergames for persons living with dementia: User-centered design study as a multistakeholder team during the COVID-19 pandemic. JMIR Serious Games 2022;10(1):e29987; doi: 10.2196/29987 35044320 PMC8772876

[11] Papaioannou T, Voinescu A, Petrini K, et al. Efficacy and moderators of virtual reality for cognitive training in people with dementia and mild cognitive impairment: A systematic review and meta-analysis. J Alzheimers Dis 2022;88(4):1341–1370; doi: 10.3233/JAD-210672 35811514

[12] Zuschnegg J, Schoberer D, Häussl A, et al. Effectiveness of computer-based interventions for community-dwelling people with cognitive decline: A systematic review with meta-analyses. BMC Geriatr 2023;23(1):229; doi: 10.1186/s12877-023-03941-y 37041494 PMC10091663

[13] Qiu R, Gu Y, Xie C, et al. Virtual reality-based targeted cognitive training program for Chinese older adults: A feasibility study. Geriatr Nurs 2022;47:35–41; doi: 10.1016/j.gerinurse.2022.06.007 35839753

[14] Perra A, Riccardo CL, De Lorenzo V, et al. Fully immersive virtual reality-based cognitive remediation for adults with psychosocial disabilities: A systematic scoping review of methods intervention gaps and meta-analysis of published effectiveness studies. Int J Environ Res Public Health 2023;20(2):1527; doi: 10.3390/ijerph20021527 36674283 PMC9864668

[15] Brooke J. SUS: A “quick and dirty” usability scale. In: Usability evaluation in industry. (Jordan PW, Thomas B, Weerdmeester BA, McClelland IL., eds.) Taylor & Francis: London; 1996, pp. 189–194. Available from: 10.1201/9781498710411-35

[16] Bangor A, Kortum PT, Miller JT. An empirical evaluation of the system usability scale. Int J Hum Comput Interact 2008;24(6):574–594; doi: 10.1080/10447310802205776

[17] Harada CN, Love MC, Triebel K. Normal cognitive aging. Clin Geriatr Med 2013;29(4):737–752; doi: 10.1016/j.cger.2013.07.002 24094294 PMC4015335

[18] Sherman DS, Mauser J, Nuno M, et al. The efficacy of cognitive intervention in mild cognitive impairment (MCI): A meta-analysis of outcomes on neuropsychological measures. Neuropsychol Rev 2017;27(4):440–484; doi: 10.1007/s11065-017-9363-3 29282641 PMC5754430

[19] Wu J, Ma Y, Ren Z. Rehabilitative effects of virtual reality technology for mild cognitive impairment: A systematic review with meta-analysis. Front Psychol 2020;11:1811; doi: 10.3389/fpsyg.2020.01811 33101098 PMC7545425

[20] Ge S, Zhu Z, Wu B, et al. Technology-based cognitive training and rehabilitation interventions for individuals with mild cognitive impairment: A systematic review. BMC Geriatr 2018;18(1):213–219; doi: 10.1186/s12877-018-0893-1 30219036 PMC6139138

[21] Hung SC, Ho AY, Lai IH, et al. Meta-analysis on the effectiveness of Virtual Reality Cognitive Training (VRCT) and Computer-Based Cognitive Training (CBCT) for individuals with Mild Cognitive Impairment (MCI). Electronics 2020;9(12):2185; doi: 10.3390/electronics9122185

[22] Thapa N, Park HJ, Yang JG, et al. The effect of a virtual reality-based intervention program on cognition in older adults with mild cognitive impairment: a randomized control trial. J Clin Med 2020;9(5):1283; doi: 10.3390/jcm9051283 32365533 PMC7288029

[23] Lawton MP, Brody EM. Assessment of older people: Self-maintaining and instrumental activities of daily living. Gerontologist. 1969;9(3):179–186; doi: 10.1093/geront/9.3_Part_1.179 5349366

